# Educational strategies for enhancing medical students’ competency in laboratory medicine practice: a scoping review

**DOI:** 10.3389/fmed.2026.1799809

**Published:** 2026-05-11

**Authors:** Yonggang Yang, Shuihua Xu, Baobing Chen, Song Chen, Wei Xu, Lijia Lu, Xiurong Zhu, Hongqiang Liao, Jiyun Tian

**Affiliations:** 1 Department of Clinical Laboratory, Hangzhou Third People’s Hospital, Hangzhou, Zhejiang, China; 2 Department of Dentistry, Tianshui Wulin Subdistrict Community Health Service Center, Hangzhou, Zhejiang, China

**Keywords:** clinical competence, educational strategies, laboratory medicine education, medical students, scoping review

## Abstract

**Background:**

Laboratory medicine supports approximately 70% of clinical decisions, yet its integration into undergraduate medical education remains inadequate, leading to deficiencies in medical students’ ability to interpret and apply laboratory results in clinical practice.

**Objectives:**

This scoping review aimed to systematically identify and describe educational strategies designed to enhance clinical medical students’ competency in laboratory medicine practice, and to analyze their instructional characteristics and implementation contexts.

**Methods:**

Following the Arksey and O’Malley framework and PRISMA-ScR guidelines, we searched PubMed, WOS, CNKI, and other databases (2015–2025) for relevant studies in Chinese and English. Two reviewers independently screened and extracted data. Descriptive statistics and inductive qualitative content analysis were used for synthesis.

**Results:**

Eleven studies were included, primarily from China, Iran, the USA, and others. Educational strategies were synthesized into three themes: (1) structured integration with clinical workflows; (2) technology-enhanced interaction and simulation; and (3) collaborative learning and authentic workplace engagement. Strategies were often used in combination, with effectiveness influenced by curricular stage and institutional resources. Existing research predominantly focused on result interpretation, with limited attention to test selection, critical value management, and interprofessional communication.

**Conclusion:**

This review provides a systematic mapping of educational strategies for developing competency in laboratory medicine, highlighting gaps in study design, long-term outcome evaluation, and coverage of key competency domains. The findings provide a preliminary mapping of current educational strategies, highlight significant gaps in study design and competency coverage, and offer directions for future research in this critical area of medical education.

## 1 Introduction

Laboratory medicine serves as the cornerstone of modern clinical practice, with an estimated 70% of clinical decisions relying on the objective data it provides (1). Despite its indispensable role in clinical decision-making, multiple studies indicate that its integration into undergraduate medical education remains inadequate, representing a well-documented educational deficit (2, 3). This disconnect between theory and clinical application directly results in significant gaps in medical students’ core competencies, including test selection, result interpretation, and clinical integration (4, 5).

The concept of Value-Based Laboratory Medicine (VBLM) has recently gained increasing attention, emphasizing that the ultimate goal of laboratory testing is not merely analytical accuracy, but the improvement of patient outcomes and healthcare sustainability (6, 7). Within this framework, laboratory professionals are expected to act as consultants who ensure the right test for the right patient at the right time, and to transform laboratory data into actionable clinical information (8). However, the education of future physicians in laboratory medicine has not kept pace with this paradigm shift. Preanalytical errors—such as inappropriate test selection, incorrect sample collection, and misinterpretation of results—remain a major source of diagnostic errors and patient harm (9–11). Studies have shown that up to 15% of preanalytical errors can lead to moderate to severe patient outcomes (12), and that many of these errors are preventable through better education and training (13).

Furthermore, emerging evidence underscores the critical link between laboratory professionals’ competency and patient safety. Credentialed laboratory scientists have been shown to make significantly fewer errors than non-certified personnel, highlighting the importance of structured education and certification pathways (13, 14). The increasing complexity of laboratory diagnostics—including the rise of point-of-care testing, digitalization, and artificial intelligence—has transformed modern healthcare (15). However, medical students often receive fragmented or insufficient training in laboratory medicine, contributing to preventable errors in clinical practice (16)

In response to this challenge, a broad consensus has emerged within the medical education community (3), leading to the establishment of corresponding graduate competency standards (17, 18). In recent years, educators have explored various teaching models, such as Case-Based/Problem-Based Learning (CBL/PBL), integration of clinical diagnostic pathways, and the application of digital tools. However, existing related studies exhibit considerable heterogeneity in intervention design, target populations, and evaluation methods, resulting in fragmented evidence. Therefore, there is a pressing need for a systematic scoping and synthesis of existing educational interventions. This study aims to systematically identify and describe educational intervention strategies reported in the literature that are designed to enhance laboratory medicine practice competency among clinical medical students. It will focus on analyzing how these strategies are centered on clinical practice and will elucidate their teaching methodologies, implementation contexts, targeted competency domains, and related effectiveness evidence. By mapping the existing research, this review intends to construct a knowledge map of the field, providing a preliminary reference for future curriculum design, teaching reforms, and research directions while acknowledging the current limitations in evidence quality and scope.

## 2 Methods

### 2.1 Protocol

This scoping review employed a collaborative team approach, guided by the established methodological framework for scoping reviews proposed by Arksey and O’Malley (19) and further enhanced by the subsequent clarifications and recommendations provided by the Joanna Briggs Institute (JBI) (20, 21). The reporting will adhere to the Preferred Reporting Items for Systematic reviews and Meta-Analyses extension for Scoping Reviews (PRISMA-ScR) checklist (22) (see Supplementary Material 1 for the PRISMA checklist). The PCC (Population, Concept, Context) framework guided the review process to ensure clarity and validity of the objectives and inclusion criteria (20, 21). Our protocol was registered on the International Platform of Registered Systematic Review and Meta-Analysis Protocols (INPLASY) under registration number: INPLASY20251200, doi: 10.37766/inplasy2025.12.0034 (see Supplementary Material 2). Searches were conducted in English for English-language databases and in Chinese for Chinese-language databases. We used artificial intelligence software, specifically DeepL Translator (version 4.5), for translation between English and non-English languages, as this tool has been shown to be highly reliable in academic contexts (23). As this is a scoping review, it does not include a formal quality assessment of the studies or a meta-analysis.

### 2.2 Inclusion and exclusion criteria

#### 2.2.1 Inclusion criteria

Guided by the PCC framework (19–21), studies meeting all the following criteria were included:

(1) Population: The review included studies focusing on clinical medical students, i.e., individuals enrolled in undergraduate (pre-doctoral) medical degree programs aimed at qualifying as physicians. This may include medical students at stages of clinical coursework, clinical skills training, and clerkships/rotations.
(2) Concept: The core concept was educational strategies aimed at cultivating competency in laboratory medicine practice (i.e., abilities in test selection, result interpretation, clinical integration, and decision-making). This encompassed any described teaching interventions, methods, curriculum components, instructional approaches, or tools (e.g., specific courses, modules, workshops, e-learning, simulation, bedside teaching, clinical reasoning exercises). The strategy must have explicitly aimed to enhance knowledge, skills, or attitudes directly related to the clinical application of laboratory medicine. This includes, but is not limited to: test selection/appropriateness, result interpretation, diagnostic reasoning, understanding pre-/post-analytical factors, and the effective utilization of laboratory data in clinical decision-making.
(3) Context: Formal medical education settings. This included educational activities conducted in medical schools, teaching hospitals, or affiliated clinical training sites as part of the core medical degree program.

#### 2.2.2 Exclusion criteria

Studies meeting any of the following conditions were excluded:

(1) Studies focusing solely on postgraduate trainees (e.g., residents, fellows), nursing students, or students of related health professions (e.g., clinical laboratory science students).
(2) Studies whose educational content involved only the theoretical instruction or operational details of laboratory techniques, without an explicit aim to cultivate their application competency in clinical diagnosis and therapeutic decision-making.
(3) Studies conducted within the context of Continuing Professional Development (CPD) or pure workplace training targeted at qualified personnel.
(4) Literature for which the full text was unavailable through any channel during the full-text screening stage.
(5) Literature not published in Chinese or English.

### 2.3 Types of evidence

The scope of searching for a scoping review should be determined based on its specific objectives (20). Given that the core aim of this review is to identify, describe, and conduct a thematic analysis of published educational intervention strategies with accessible, detailed methodological information, our search focused on major peer-reviewed literature databases. Literature types included, but were not limited to: quantitative or qualitative primary studies (such as Randomized Controlled Trials (RCTs), cohort studies, pre-post studies, cross-sectional surveys, qualitative research), curriculum or teaching program evaluation reports, systematic reviews, and scoping reviews. For perspective articles (e.g., commentaries, letters, editorials), full-text assessments were performed; they were included only if they provided detailed descriptions, analyses, or framework recommendations concerning specific educational strategies or curriculum models. Articles expressing only general opinions or engaging in simple discussions were excluded. While we acknowledge the value of gray literature (e.g., conference abstracts, dissertations), the preliminary search for this review did not include sources of gray literature to ensure the accessibility of detailed methods and results from included studies for in-depth thematic analysis.

### 2.4 Search strategy and study selection

We developed the search strategy following the PRISMA-S extension guidelines. Initially, three core conceptual blocks were identified through team discussions and a review of key literature: (1) clinical medicine education, (2) laboratory medicine, and (3) education/training. Subsequently, preliminary searches were conducted in PubMed and Web of Science. By analyzing high-frequency keywords and MeSH terms from the search results and verifying that five known highly relevant articles (“seed articles”) could be retrieved, we iteratively refined the search term list.

Two authors independently screened five databases: PubMed, Web of Science, China National Knowledge Infrastructure (CNKI), Springer LINK, and the Chinese Medical Association (CMA) journals database. The search was performed from April to July 2025. The search strategy was adapted according to the characteristics of each database: in Chinese databases (CNKI, CMA), Chinese subject headings or the abstract field were primarily used; in English-language databases (PubMed, Web of Science, Springer LINK), a combination of MeSH terms and title/abstract free-text words was employed to ensure sensitivity and precision. Further manual searching was conducted, including screening the reference lists of relevant published systematic reviews or meta-analyses. Detailed search strings for each database are provided in Supplementary Material 3.

This review searched for literature published between 1 January 2015, and 30 June 2025. The rationale for this timeframe is 2-fold: since around 2015, global medical education has comprehensively shifted toward Competency-Based Medical Education (CBME), with the teaching core moving from knowledge transmission to the systematic cultivation of clinical practice competencies (24); this starting point ensures the review focuses on strategies relevant to the modern educational paradigm. Since the mid-2010s, the application of digital technologies such as virtual simulation and mobile learning in medical education has rapidly proliferated, providing innovative tools for the practical teaching of laboratory medicine (25); this timeframe can effectively capture key evidence of this rapid integration of educational technology. The search was not restricted by language of publication (but final screening was limited to Chinese and English literature).

In summary, this timeframe was set to systematically synthesize the latest evidence that aligns with current mainstream educational philosophies and technological advancements. According to the inclusion and exclusion criteria, search results from all databases were imported into the reference management software EndNote (version 25.0, Clarivate Analytics) for merging, and its deduplication function was used to automatically remove duplicate records. Subsequently, the unique records after deduplication were imported into the systematic review management software Covidence (Veritas Health Innovation, Melbourne, Australia) for screening.

The screening process was divided into two stages:

Title and abstract screening based on PCC criteria: Two researchers independently reviewed all records and made judgments based on the pre-established inclusion/exclusion criteria (PCC framework). Any record deemed potentially eligible by either researcher proceeded to the next stage.
Full-text screening: Full texts of the records included in the previous stage were obtained. The same two researchers then independently reviewed the full texts to determine final eligibility. At this stage, records for which the full text was unavailable were excluded, and the reason was documented.

At each screening stage, the judgments of the two researchers were cross-checked. Any disagreements were first resolved through discussion between the two researchers. If consensus could not be reached after discussion, a third senior researcher was consulted for arbitration. The entire screening process was designed to be transparent and reproducible.

The selection process is detailed in the PRISMA-ScR flow diagram (see Supplementary Material 4).

### 2.5 Data extraction and charting

Corresponding author Jiyun Tian pre-designed a standardized data extraction form in Microsoft Excel based on the review objectives and the PCC framework (20, 21) (see Supplementary Material 5) to ensure consistency among reviewers. The form aimed to capture data relevant to evidence mapping, rather than extracting detailed results for effect synthesis, which aligns with the purpose of a scoping review (20, 21, 26). Preliminary charting categories included: (1) Study identification and context: authors (year), country, source of evidence, study objective; (2) Participants: target learner population; (3) Concept: details of the educational strategy/intervention (strategy description, teaching methodology, mode of implementation, duration); (4) Context: educational setting (e.g., university, teaching hospital); (5) Evaluation methodology: research design, tools employed, types of outcomes reported (e.g., knowledge, skills, attitudes); (6) Key findings and conclusions: reported results, strengths, limitations, and recommendations proposed by the authors.

This form underwent pilot testing on a sample of included sources (e.g., 2–3 articles) by at least two independent reviewers and was iteratively refined based on team discussions (21). Data extraction was then conducted independently by two reviewers. Throughout the extraction process, regular team meetings were held to discuss progress, resolve issues, and ensure the form captured all necessary information. The finalized data extraction form is available in Supplementary Material 5.

### 2.6 Data analysis and synthesis strategy

The analysis followed a structured multi-stage approach, combining quantitative and qualitative descriptive methods to map the evidence, in accordance with JBI recommendations (20, 21). The synthesis did not include meta-analysis, meta-synthesis, or critical appraisal of the risk of bias in the evidence, as these are beyond the scope of a scoping review (20, 26, 27).

#### 2.6.1 Quantitative descriptive analysis

The basic characteristics of the included studies were described and presented as percentages. This included the temporal and geographical distribution of the evidence, research design methods, the stage of the target learners, and the focal points of laboratory medicine practice competencies addressed.

#### 2.6.2 Basic qualitative content analysis

This study employed an inductive qualitative content analysis method to construct themes from the extracted data on “educational intervention strategies,” strictly adhering to JBI methodological guidance for scoping reviews.

Team members thoroughly read the “core description of educational strategies” text for all included studies in the data extraction table. Two researchers independently performed open coding on each strategy description, using short words or phrases to capture their instructional features (e.g., “use of decision support tools,” “small group case analysis,” “following standardized pathways,” “online gamified learning,” “interprofessional collaborative discussion”). Subsequently, the two researchers met to compare and discuss the independently generated codes, organizing codes with similar meanings to form preliminary categories. Microsoft Excel was used throughout this process to manage codes and facilitate the organization of preliminary categories. The research team then further abstracted, compared, and integrated these preliminary categories, distilling them into higher-level themes based on their inherent relationships. The naming of each theme aimed to accurately summarize the common essential characteristics of all strategies under it. Through team validation and iteration, consensus on the thematic structure was reached, mapping all educational strategies from the included literature to form a clear knowledge map.

#### 2.6.3 Narrative synthesis of implementation context and evidence gaps

Through an in-depth interpretation of the extracted data, the following two objectives were achieved: analyzing the interaction between educational strategies and the specific contextual factors they rely on (regional and systemic differences, applicability to educational stages, institutional and resource backgrounds, depth and mode of integration); and identifying the deficiencies and future directions in the research field by critically examining the distribution, quality, and comprehensiveness of the existing evidence.

## 3 Results

### 3.1 Study selection

The literature screening was conducted in accordance with the PRISMA-ScR guidelines. The initial search yielded 9,946 records, comprising 7,613 from PubMed, 77 from Web of Science, 1,485 from CNKI, 212 from Springer LINK, and 556 from the Chinese Medical Association (CMA) database. Additionally, three records were identified through reference tracing of relevant systematic reviews. After removing 641 duplicate records, a total of 9,305 unique records underwent title and abstract screening. At this stage, 9,067 records were excluded as they did not meet the inclusion criteria based on the PCC framework.

Subsequently, full texts of the remaining 238 articles were retrieved for eligibility assessment. Following full-text review, a further 227 articles were excluded. The main reasons for exclusion were: Ineligible population (*n* = 101, 44.5%), Non-practical educational intervention (*n* = 78, 34.4%), Irrelevant to the topic (*n* = 43, 18.9%), Full text unavailable (*n* = 3, 1.3%) and non-Chinese/English language (*n* = 2, 0.9%). A detailed breakdown of exclusion reasons with corresponding references is provided in Supplementary Material 4.

Ultimately, 11 studies were included in this scoping review. The entire screening process was performed independently by two researchers, and any disagreements were resolved through discussion or consultation with a third senior researcher. The study selection flow is detailed in Supplementary Material 4.

### 3.2 Quantitative descriptive analysis—characteristics of included studies

**FIGURE 1:**
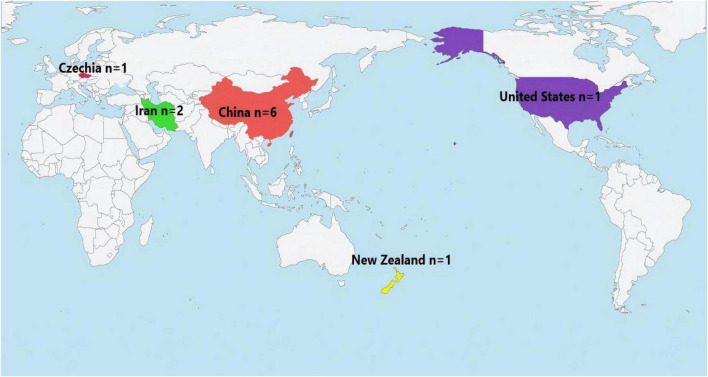
Geographical distribution of included studies (*n* = 11).

Through systematic search and screening, 11 relevant articles were ultimately included in this review (five from the CMA, 1 from CNKI, two from PubMed, one from WOS and two from reference lists). The publication years of the included studies spanned from 2017 to 2024. Among these, a total of seven studies were published in the recent 5 years (2020–2024), indicating sustained interest in this field. China was the primary source of the research output, contributing six studies. The remaining five studies were from Iran (2), the United States, the Czech Republic, and New Zealand (one each) [see Figure 1 for the geographical distribution of included studies (*n* = 11)]. One study (28) involved authors from different healthcare professional backgrounds, reflecting interprofessional collaboration. The included literature comprised six Chinese and five English articles. The research designs were diverse, including non-RCTs (seven studies), RCTs (two studies), descriptive studies (program reports and preliminary evaluations, one study), and analytical studies (cross-sectional survey, one study). All included studies were primary research; no review articles were included. The main characteristics are summarized in Supplementary Material 6 for details.

Table 1 presents the detailed characteristics of the included studies. Beyond the basic characteristics reported previously, we assessed the completeness of key information reporting across studies. Only three studies (27.3%) explicitly reported students’ prior knowledge or experience in laboratory medicine (29–31). Intervention duration was clearly described in seven studies (63.6%) (28–34), while the remaining four studies (46.4%) provided insufficient detail to determine the intensity or frequency of the educational intervention. Furthermore, none of the included studies reported long-term follow-up outcomes (>6 months), limiting conclusions about the sustainability of observed effects.

**TABLE 1:** Basic characteristics of included studies (*n* = 11) with reporting completeness.

Characteristic	Category	*n* (%)	Reference number(s)
Basic study characteristics			
Publication year	2017–2019	4 (36.4)	(29, 30, 32, 36)
	2020–2024	7 (63.6)	(28, 31, 33–35, 37, 38)
Country/region	China	6 (54.5)	(33–38)
	Iran	2 (18.2)	(29, 30)
	Czechia, USA, New Zealand	1 each (9.1 each)	(31, 33, 35)
Research design	Non-RCT (quasi-experimental)	7(63.6)	(29–32, 34, 36, 37)
	RCT	2(18.2)	(33, 35)
	Descriptive study (Program report and preliminary evaluation)	1(9.1)	(28)
	Analytical study (Cross-sectional survey)	1(9.1)	(38)
Target learner stage	Pre-clinical/clerkship	8 (72.7)*	(29–34, 37, 38)
	Clinical internship	5 (45.5)*	(28–30, 35, 36)
Core competency focus	Analysis and application of test indicators	9 (81.8)	(29–32, 34–38)
	Pre-analytical quality control	1 (9.1)	(33)
	Interprofessional collaboration and communication	1 (9.1)	(28)
Reporting completeness indicators			
Prior knowledge reported	Yes	3 (27.3)	(29–31)
	No	8 (72.7)	28, (32–38)
Intervention duration Reported	Clearly described	7 (63.6)	(28–34)
	Insufficient detail	4 (36.4)	(35–38)
Long-term Follow-up (>6 months)	Yes	0 (0)	–
	No	11 (100)	(28–38)

Percentages for target learner stage may sum to >100% as some studies included multiple learner stages. Reporting completeness indicators assess whether studies provided explicit information about these methodological details. The symbol * indicates that the percentages for target learner stage may sum to more than 100% because some studies included multiple learner stages.

Regarding study design quality, of the 11 included studies, 2 (18.2%) were randomized controlled trials (33, 35), 7 (63.6%) were non-randomized comparative studies (29–32, 34, 36, 37), others were 1 descriptive study (28) and one analytical study (38). This predominance of non-randomized designs indicates that the evidence base is largely derived from lower-quality study designs, warranting cautious interpretation of reported effects.

Furthermore, we systematically reviewed the measurement tools employed in the included studies and their distribution. Table 2 summarizes the types of assessment instruments used across the 11 studies, including questionnaires, knowledge/skill tests, qualitative feedback, and practical performance evaluations.

**TABLE 2:** Distribution of assessment tools across included studies (*n* = 11).

Assessment tool category	*n* (%)	Reference number(s)
Questionnaires	10 (90.9)	(28–31, 33–38)
Teaching satisfaction	9 (81.8)	(28–31, 33, 34, 36–38)
Learning interest and motivation	4 (36.4)	(28, 29, 31, 33)
Self-perceived ability enhancement	3 (27.3)	(28, 29, 31)
Perception of course necessity and value	2 (18.2)	(30, 31)
Confidence improvement	2 (18.2)	(28, 31)
Knowledge/skill tests	8 (72.7)	(29, 31–37)
Qualitative feedback	4 (36.4)	(28, 29, 31, 32)
Practical performance evaluation	1 (9.1)	(33)

The sum of subcategory counts for questionnaires exceeds 10 because individual studies often assessed multiple dimensions within a single questionnaire.

Questionnaires were the most common evaluation method, applied in 10 studies (90.9%) to assess attitudes, perceptions, or satisfaction (28–31, 33–38). As shown in Table 2, these questionnaires covered multiple assessment dimensions, with teaching satisfaction being the most frequently measured outcome (nine studies, 81.8%), followed by learning interest and motivation (four studies, 36.4%), students’ self-perceived ability enhancement (three studies, 27.3%), perception of course necessity and value (two studies, 18.2%), and confidence improvement (two studies, 18.2%).

Knowledge and skill tests were used in eight studies (72.7%) (29, 31–37), with formats including standardized examinations, case analysis questions, in-class quizzes, and specific skill assessment forms (e.g., interpretation skill evaluation). These tests aimed to directly assess students’ mastery of theoretical knowledge and clinical reasoning skills related to laboratory medicine.

Qualitative feedback was collected in four studies (36.4%) (28, 29, 31, 32) through open-ended questions or post-course comments, serving as a supplement to quantitative results and providing insights into students’ experiences with, preferences for, and suggestions on the teaching strategies. In addition, only one study (9.1%) (33) employed practical performance evaluation, which involved assessing students’ in-class practical activities and assignments using a structured rating form to evaluate their operational competence and comprehensive application level.

### 3.3 Qualitative content analysis—thematic construction of educational intervention strategies

This section systematically synthesized and interpreted the core logic and characteristics of the educational strategies found in the included literature. Using inductive qualitative content analysis, three overarching core themes were ultimately derived: structured integration with clinical workflows, technology-enhanced interaction and situational simulation, and collaborative communities with authentic workplace engagement.

To provide a quantitative overview of the thematic distribution, Table 3 presents the number and percentage of studies contributing to each theme and its subordinate strategy categories. The analysis revealed that current educational strategies are not characterized by the application of singular methods but rather exhibit a composite nature. For instance, a single intervention might simultaneously incorporate pathway mapping (Theme 1, Category 1.1) and small-group case discussions (Theme 1, Category 1.3). Together, these three themes constitute a multi-dimensional strategic framework that progresses from the restructuring of knowledge, through the optimization of the learning process, to immersion in authentic practice contexts.

**TABLE 3:** Thematic categories and distribution of educational strategies across included studies (*n* = 11).

Theme and category	*n* (%)	Reference number(s)
Theme 1: structured integration with the clinical workflows	9 (81.8)	(28, 30, 32–38)
1.1 Application of clinical laboratory diagnostic pathway maps	2 (18.2)	(34, 37)
1.2 Use of standardized analytical decision tools	1 (9.1)	(35)
1.3 CBL/PBL	6 (54.5)	(28, 32, 33, 35, 36, 38)
1.4 Curriculum content expansion and restructuring	1 (9.1)	(30)
Theme 2: technology-enhanced interaction and situational simulation	2 (18.2)	(31, 32)
2.1 Simulation and problem-solving based on digital platforms	2 (18.2)	(31, 32)
2.2 Design and application of gamified online learning modules	1 (9.1)	(31)
Theme 3: collaborative communities and authentic workplace engagement	3 (27.3)	(28, 29, 33)
3.1 Interprofessional collaborative learning	1 (9.1)	(28)
3.2 Firsthand experience in practical settings	2 (18.2)	(29, 33)

Theme and category represent the three core themes derived from qualitative content analysis and their subordinate specific strategy categories. *n* (%) indicates the number and percentage of the 11 included studies that involved each strategy category. The sum of subcategory counts exceeds 11 because a single study could describe or evaluate multiple strategies. Reference number(s) correspond to the specific study numbers in the reference list supporting each strategy category [e.g., (36) represents Wang et al., 2018].

### 3.4 Narrative synthesis of implementation context and evidence gaps

This section aims to conduct an in-depth analysis of the complex interactions between educational strategies and their specific contexts, and to systematically examine the distribution characteristics and inherent limitations of the existing evidence, thereby clarifying key gaps and future directions in this field of research.

#### 3.4.1 Interaction between educational strategies and implementation context

Strategy orientation under regional and systemic differences: The included Chinese studies tended to favor deep integration and reform within the existing core curriculum framework. For example, several studies focused on developing and applying “clinical laboratory diagnostic pathways” for specific diseases (e.g., chronic hepatitis B and C) to systematically restructure the theoretical teaching content of courses like Laboratory Diagnostics (34, 37). In contrast, some international studies presented strategies designed as independent or supplementary modules, such as gamified online pre-class modules for flipped classrooms (31), short-term intensive laboratory rotation courses (29), or pilot Interprofessional Education (IPE) projects (28). These differences may reflect variations in curricular structures and reform pathways.

These differences likely reflect fundamental variations in medical education structures and reform philosophies across regions. The Chinese approach, emphasizing integration within existing courses, may be shaped by a centralized curriculum system where core courses are standardized and modifications require institutional-level approval. Conversely, the independent module approach seen in some Western studies may reflect greater curricular flexibility and a tradition of piloting innovations before broader integration. However, it remains unclear whether one approach yields superior educational outcomes. While some evidence suggests that integrated curricula provide students with comparable opportunities to develop essential competencies across different program phases (39), direct comparisons of the effectiveness and feasibility of these two approaches in different educational contexts are lacking. The deep integration model may ensure sustainability and scalability, but risks being constrained by existing course structures and resource limitations (40). The independent module model allows for rapid innovation and evaluation, but may face challenges in long-term sustainability and integration into core curricula. Future research should directly compare the effectiveness and feasibility of these two approaches in different educational contexts.

Alignment of Educational Stage and Strategy Function: The design logic of strategies is highly related to the clinical experience level of the target learners. Strategies targeting pre-clinical or clerkship students primarily serve the core function of constructing a foundational cognitive framework and initial clinical connections. Consequently, strategies emphasizing structure, guidance, and intuitiveness were widely adopted. Examples include using clinical pathway maps for systematic explanation (34, 37), utilizing decision support tools to simplify complex judgments (35), or employing gamified online modules for pre-class guidance (31). For students in clinical internships, strategies focus more on promoting knowledge integration and clinical transfer. Therefore, immersive experiences in authentic clinical environments [e.g., practical work in clinical laboratories (33), laboratory rotations (29)], interprofessional collaborative tasks based on complex real-world scenarios (28), or high-fidelity simulations of complete workflows (32) become more prominent.

While this alignment appears intuitive and pedagogically sound, it may also reflect a publication bias or the logistical ease of implementing certain strategies at specific stages. For instance, immersive experiences are logistically more feasible during internships when students are already in clinical settings. Conversely, structured guidance tools are easier to implement in classroom settings. Whether this stage-strategy alignment is optimal for learning outcomes remains empirically under-examined. Some studies suggest that even pre-clinical students could benefit from early exposure to authentic clinical scenarios (41), and that interns might still need structured guidance for complex diagnostic reasoning (42). Future research should explore whether cross-stage strategy application yields additional benefits, and whether the observed alignment represents best practice or merely convenience. Such investigations would help determine whether the current stage-strategy alignment is pedagogically optimal or simply a reflection of implementation logistics and publication biases.

Constraints of Institutional Resources on Strategy Feasibility: The complexity and generalizability of strategies are significantly influenced by institutional resources. Highly customized and technology-intensive strategies, such as developing interactive bioinformatics teaching applications (32) or specialized forms with automated interpretation functions (35), place higher demands on technological and human resources. Similarly, the successful implementation of IPE (28) relies on stable cross-departmental collaboration mechanisms. In contrast, strategies based on modifications of classic teaching methods, such as CBL/PBL teaching centered on standard cases (38), primarily depend on teachers’ clinical knowledge base and teaching facilitation skills. They are less reliant on additional technology or inter-institutional coordination and may therefore possess better generalizability.

This observation raises important questions about educational equity and the transferability of research findings. If highly effective strategies are only feasible in resource-rich institutions, their widespread adoption may be limited, potentially widening the gap between well-resourced and under-resourced medical schools (43). Conversely, while low-resource strategies like CBL/PBL appear more generalizable, their effectiveness may be compromised without adequate faculty training or institutional support. The literature currently lacks rigorous cost-effectiveness analyses comparing these strategies (43). Moreover, the definition of “resources” should extend beyond financial and technological capital to include faculty expertise, institutional culture, and administrative support—factors that may be equally critical but harder to quantify. Future research should explicitly examine the resource requirements of educational interventions and explore adaptive implementations that maintain core pedagogical principles while accommodating local constraints.

Depth and models of curriculum integration: The ways in which educational strategies combine with existing curricula present different models. The first is the deep integration model, where a strategy directly reshapes the content structure and instructional sequence of a core course, such as replacing traditional lectures with “disease-specific clinical laboratory diagnostic pathways” (34, 37). The second is the parallel supplementary model, where a strategy exists as an independent short-term course (29), an extracurricular activity (28), or a pre-class component for a flipped classroom (31).

These two models represent different philosophical approaches to curriculum innovation. The deep integration model assumes that laboratory medicine education should be woven into the fabric of clinical training, emphasizing its relevance to routine practice. The parallel model treats laboratory medicine as a distinct competency requiring dedicated focus. Evidence suggests that integrated curricula can provide students with comparable opportunities to develop essential competencies across different program phases (39); however, their implementation may be constrained by resource limitations and existing course structures (40).

Neither model has been definitively shown to be superior. Deep integration may risk the content becoming diluted or overshadowed by other clinical topics. Parallel modules may struggle with transfer of learning to clinical practice. Importantly, these models are not mutually exclusive; some institutions may benefit from a hybrid approach that combines integrated teaching with focused modules. Comparative effectiveness research is urgently needed to inform this decision-making process. Studies comparing different educational interventions, such as the evaluation of a virtual patient curriculum for dizziness diagnosis (44), demonstrate the feasibility and value of such rigorous comparisons in assessing clinical reasoning outcomes.

#### 3.4.2 Evidence gaps and future research directions

This study systematically synthesized the existing literature, revealing significant limitations in the field regarding research methodology, content coverage, and theoretical depth.

Limitations in research methodology: The primary weakness of the current body of evidence lies in the insufficient rigor of study designs. The majority of interventional studies (7/11) employed non-randomized designs [e.g., historical controls (29), non-randomized concurrent controls (30)], rendering their conclusions susceptible to confounding factors and limiting causal inferences. This highlights the need for adherence to reporting guidelines such as STROBE (45) to enhance transparency and quality. There is a pressing need for more rigorously designed RCTs to provide higher-level evidence (38). More critically, all included studies assessed only immediate or short-term effects, lacking long-term longitudinal follow-up data. This precludes evaluation of the potential impact of educational interventions on lasting changes in clinical behavior. This finding aligns with suggestions from multiple authors for the need to conduct long-term follow-up and multi-center studies (29).

Imbalance in the Scope of Practical Competency Development: Existing research is highly concentrated on cultivating competency in test result interpretation and diagnostic reasoning within specific disease contexts. However, significant gaps remain in research on developing several other key competencies for clinical practice, including: (1) The evidence-based selection and value consideration of laboratory tests, a core competency for the rational application of laboratory medicine that has not yet become a curriculum focus (30); (2) The clinical recognition of critical values and the corresponding emergency response protocols-an area entirely absent from the included studies despite its importance for patient safety; (3) Strategies and skills for efficient, standardized communication with the laboratory department. Although the cultivation of interprofessional collaboration competency has received attention (28), its depth and breadth require expansion, particularly concerning specific communication strategies.

Lack of Theoretical Grounding and Exploration of Contextual Mechanisms: Most studies failed to clearly articulate the pedagogical or cognitive science theoretical frameworks underpinning their instructional designs, with only a few exceptions (31). Concurrently, existing literature seldom delves into analyzing how contextual factors—such as institutional culture, faculty expertise, and differences in students’ prior knowledge—specifically moderate the effectiveness of strategy implementation. This severely limits the accurate assessment of a strategy’s generalizability across different institutions and cultural settings. Future research needs to strengthen the investigation of these contextual mechanisms.

Scarcity of research on personalized learning and educational equity: The vast majority of reported teaching strategies employ a “one-size-fits-all” approach, with only one study explicitly exploring a personalized clerkship teaching plan for clinical medical students (38). This gap is particularly concerning given the growing recognition of the importance of individualized learning pathways in medical education, which can accommodate students’ differing learning needs, paces, and circumstances, thereby promoting both educational effectiveness and equity (46, 47). Research has shown that students vary considerably in their approaches to learning in clinical settings, and those with stronger self-regulated learning skills are better able to maximize learning opportunities (48). Furthermore, the field entirely lacks investigation into the differential effects of educational interventions—that is, whether the same teaching strategy yields heterogeneous effects on students with different academic backgrounds, clinical experience, or learning styles. Future instructional design and research should incorporate greater consideration of learners’ individual characteristics to promote educational equity and optimize effectiveness.

## 4 Discussion

Through this systematic scoping review, we have integrated, for the first time, diverse educational strategies to construct a conceptual map. By analyzing 11 studies, we have identified three core strategic themes, revealed their context-dependent nature, and highlighted major gaps within the current evidence base. The following sections will summarize the main findings and limitations of this review and propose specific recommendations for educational practice, policy, and research.

### 4.1 Main findings

This scoping review has systematically identified and described educational strategies targeting the improvement of laboratory medicine practice competency among clinical medical students. We found that current educational strategies in laboratory medicine exhibit a distinct composite nature: active learning strategies, particularly CBL/PBL, dominate over traditional didactic instruction, reflecting a shift toward competency development. Technological tools (e.g., gamification, simulation) primarily serve as enabling means to enhance the interactivity and contextual authenticity of traditional teaching methods. Furthermore, educational design emphasizes the fusion of “structuration” and “contextualization”—providing a standardized knowledge framework through clinical pathway teaching, while embedding knowledge into authentic workplace contexts through hands-on practice and interprofessional learning. These findings collectively confirm a prevailing trend towards blended instructional design in these strategies.

The research corroborates that in response to the long-standing issues of weak laboratory medicine instruction and its disconnect from clinical practice in medical education (2, 4), educators globally have actively developed and implemented various interventions centered on competency development (3). These strategies are not fragmented or isolated; rather, they collectively construct a progressive, multi-dimensional cultivation framework that moves from restructuring the knowledge system, to optimizing the learning process, and finally to integrating into practice contexts. This framework responds to the core tenets of CBME (24).

First, the strategies under the theme of “Structured Integration with Clinical Workflows” directly address the drawback of “knowledge fragmentation” resulting from the traditional teaching approach organized by laboratory technique categories. For instance, by developing and applying “clinical laboratory diagnostic pathway maps” that span the entire diagnostic and therapeutic cycle of specific diseases (e.g., chronic viral hepatitis), test items scattered across various subspecialties are systematically connected and logically reconstructed (34, 37). This type of strategy provides students with a cognitive map isomorphic to real-world clinical workflows. From a cognitive load theory perspective, this approach reduces extraneous cognitive load by organizing information in a familiar, clinically-relevant schema, thereby freeing up working memory capacity for genuine learning and problem-solving (49, 50). Schema acquisition, as the primary mechanism distinguishing expert from novice performance, is facilitated when instructional designs minimize extraneous processing demands (49). The structured presentation of diagnostic pathways enables learners to recognize problem states as belonging to particular categories that require specific clinical moves, a process central to the development of clinical expertise.

Moreover, the cognitive benefits of structured information presentation extend beyond initial learning. Recent evidence suggests that well-organized instructional materials can enhance the accuracy of students’ cognitive load assessments by reducing anchoring biases that may distort self-reported cognitive load in complex, multi-task learning environments (51). When learners encounter clinically-relevant information in a coherent, pathway-based format, they are better able to accurately gauge their own cognitive processing demands, which in turn supports more effective self-regulated learning (52). Simultaneously, developing and applying specialized tools embedded with clinical decision logic (e.g., Excel templates for arterial blood gas interpretation) externalizes and standardizes complex, implicit analytical processes (e.g., the “four-step analysis method”), guiding students to follow clear steps in their reasoning. This effectively reduces cognitive load and minimizes operational errors (35). Together, these two approaches work toward transforming isolated bits of laboratory knowledge into a structured knowledge system applicable to solving clinical problems. Across the nine studies addressing structured integration, outcomes were primarily assessed through knowledge tests (seven studies, 77.8%) and student satisfaction questionnaires (six studies, 66.7%), with only two studies (22.2%) including skills assessment. All evaluations were conducted immediately post-intervention, with no long-term follow-up reported.

Second, the theme of “Technology-Enhanced Interaction and Situational Simulation” demonstrates the crucial role of digital technology in deepening the learning experience. On one hand, by developing highly realistic interactive online applications that simulate the complete diagnostic process from clinical consultation to laboratory analysis and finally to report interpretation (32), students learn how to utilize professional resources like bioinformatics databases to solve problems by completing a series of sequential tasks based on real data. This constructivist approach positions students as active agents who build understanding through goal-directed interaction with simulated environments (53). Simulation allows exploration and reflection in authentic contexts, facilitating the development of problem-solving schemas essential for clinical practice (53). Integrating game elements leverages gamification principles to enhance engagement and motivation (54). Gamification uses mechanics such as points, levels, and feedback to satisfy learners’ needs for competence and autonomy (54). Specific game elements correlate with improved learning: a study found significant positive correlations between learning outcomes and students’ perceived experience with feedback (*r* = 0.583), concentration (*r* = 0.509), and challenge (*r* = 0.421) (55). Thus, well-designed gamified environments should prioritize immediate feedback, maintain focus, and provide appropriately challenging tasks (55).

On the other hand, systematically integrating gamification design elements (e.g., progress feedback, points/badges, narrative scenarios) into online learning modules (31) significantly increases the appeal of learning materials and learners’ intrinsic motivation. These technology-enhanced strategies create an interactive learning environment that makes complex processes more tangible and easier to master. Both studies on technology-enhanced simulation reported positive effects on knowledge acquisition. For context, a recent meta-analysis of gamification in nursing education (56) reported a pooled effect size of 0.99 for knowledge outcomes, suggesting that gamified approaches can be effective in related health professions education settings. However, this effect size is derived from a different body of evidence and does not quantitatively represent the findings of the two studies included in this scoping review, with one study also documenting improved engagement and motivation through gamification elements (31). Assessments were conducted immediately after module completion.

Finally, the theme of “Collaborative Communities and Authentic Workplace Engagement” emphasizes the social and situated nature of learning. IPE projects intentionally form teams of students from different disciplines such as clinical medicine, Laboratory Medicine science, and nursing. These teams collaborate on complex cases involving comprehensive tasks like test ordering, result interpretation, and treatment planning (28), effectively fostering role clarification and collaborative skills among future healthcare team members. This approach aligns with situated learning theory, which posits that learning is inextricably tied to the context and activity in which it occurs (57). Through legitimate peripheral participation in communities of practice, learners gradually move from novice observers to more central members of the clinical team as they develop competence and professional identity (57, 58). Furthermore, arranging for students to enter clinical laboratories and personally participate in practical work such as specimen handling, pre-analytical processing, and quality control (29, 33) represents “learning by doing” in the most authentic workplace setting. This greatly strengthens the connection between theoretical knowledge and practical operation and enhances mutual understanding between clinical and laboratory staff. Studies on collaborative learning reported outcomes focused on interprofessional competencies (three studies) and workplace performance (two studies), assessed through self-reported questionnaires and qualitative feedback. No objective skills assessments were employed.

In summary, the three thematic strategies identified in this review signify that laboratory medicine education is transitioning from mere knowledge transmission towards a composite cultivation paradigm that emphasizes knowledge structuring, interactive learning processes, and authentic practice contexts. The synergistic application of these strategies offers a preliminary conceptual map of current approaches. However, given the limited number of studies, predominance of non-randomized designs, and absence of long-term follow-up, this map should be viewed as an early synthesis of the field rather than a validated comprehensive roadmap. It highlights promising directions while underscoring the need for more rigorous research.

### 4.2 Limitations of this review

To ensure analytical depth and feasibility, we included only peer-reviewed literature published in Chinese and English. This may have excluded valuable teaching practices from regions using other languages, introducing a potential language bias. Secondly, despite our efforts to obtain full texts, some relevant evidence may have been missed due to the unavailability of certain documents (e.g., conference abstracts or older articles from specific journals). The search for this review was primarily limited to published peer-reviewed literature and did not extensively incorporate gray literature (e.g., unpublished reports, detailed conference papers). Consequently, some ongoing or unpublished innovative teaching practices may have been omitted. Future updates to this review could consider expanding the search scope to include these sources.

Furthermore, the definitions and reporting detail of “educational strategy” varied considerably across the included studies. The interventions exhibited high heterogeneity in content, intensity, and implementation details, which limits the possibility of direct comparison between different strategies and the synthesis of their effects. Finally, as a scoping review, this study did not conduct a methodological quality assessment or risk of bias evaluation of the included literature. Therefore, conclusions regarding the effectiveness of the described strategies should be interpreted with caution and require validation through subsequent, more rigorous systematic reviews (26).

### 4.3 Research implications and recommendations

Implications for policy and curriculum planning: Educational administrators and curriculum committees should fully recognize that competency in laboratory medicine practice is a core component of clinical competence, not peripheral content (3), which highlights the urgency of systematically integrating laboratory medicine into undergraduate medical curricula. Concurrently, the incorporation of the composite strategies identified in this review (e.g., modules integrating pathway-based teaching, case discussions, and digital tools) into core curricula should be encouraged and supported. Necessary funding and faculty development support should also be provided for creating such educational resources.

Recommendations for educational practitioners: Instructional designers and implementers should selectively choose or combine strategies based on different teaching objectives and student stages. For junior students, priority can be given to guided strategies from the themes of “structured integration” and “technology enhancement” to build foundational knowledge and stimulate interest. For senior or internship-phase students, the proportion of strategies related to “workplace engagement” and “interprofessional collaboration” should be increased to facilitate the transfer of knowledge into clinical behaviors. Furthermore, when applying highly customized digital tools or implementing IPE, the institution’s resources and coordination capacity must be carefully considered to ensure the feasibility and sustainability of the intervention.

Recommendations for future research: To address the current evidence gaps, future studies should: (1) Employ more rigorous research designs, such as well-designed RCTs, and conduct long-term follow-up to assess the potential impact of educational interventions on long-term knowledge retention, clinical behaviors, and even patient care; (2) Expand the research scope beyond the focus on test interpretation for specific diseases, delving into how to effectively cultivate core practical competencies that have not received sufficient attention, such as evidence-based test selection, critical value management, and efficient consultation with laboratory departments; These competencies are integral to high-value, cost-conscious care and should be informed by principles of evidence-based laboratory medicine. Recent national initiatives and educational frameworks have emphasized the importance of integrating such competencies into graduate medical education to prepare physicians to navigate complex healthcare systems, reduce waste, and improve patient outcomes (59–61). 3) Strengthen theoretical grounding and mechanism exploration. Future research should be grounded in pedagogical or cognitive science frameworks—such as cognitive load theory, situated learning theory, or self-determination theory—to better understand why and how specific educational strategies produce their effects (49, 50, 54, 57). This would help identify the active components of interventions and improve the generalizability of findings across different educational contexts.
