# Supplementary material for: Educational strategies for enhancing medical students’ competency in laboratory medicine practice: a scoping review
Source: Front Med (Lausanne). 2026 May 11;13:1799809. doi: 10.3389/fmed.2026.1799809 (PMC13199127; doi:10.3389/fmed.2026.1799809)
Supplement: Supplementary file 1 [file Data_Sheet_1.docx]

**Supplementary material 1**


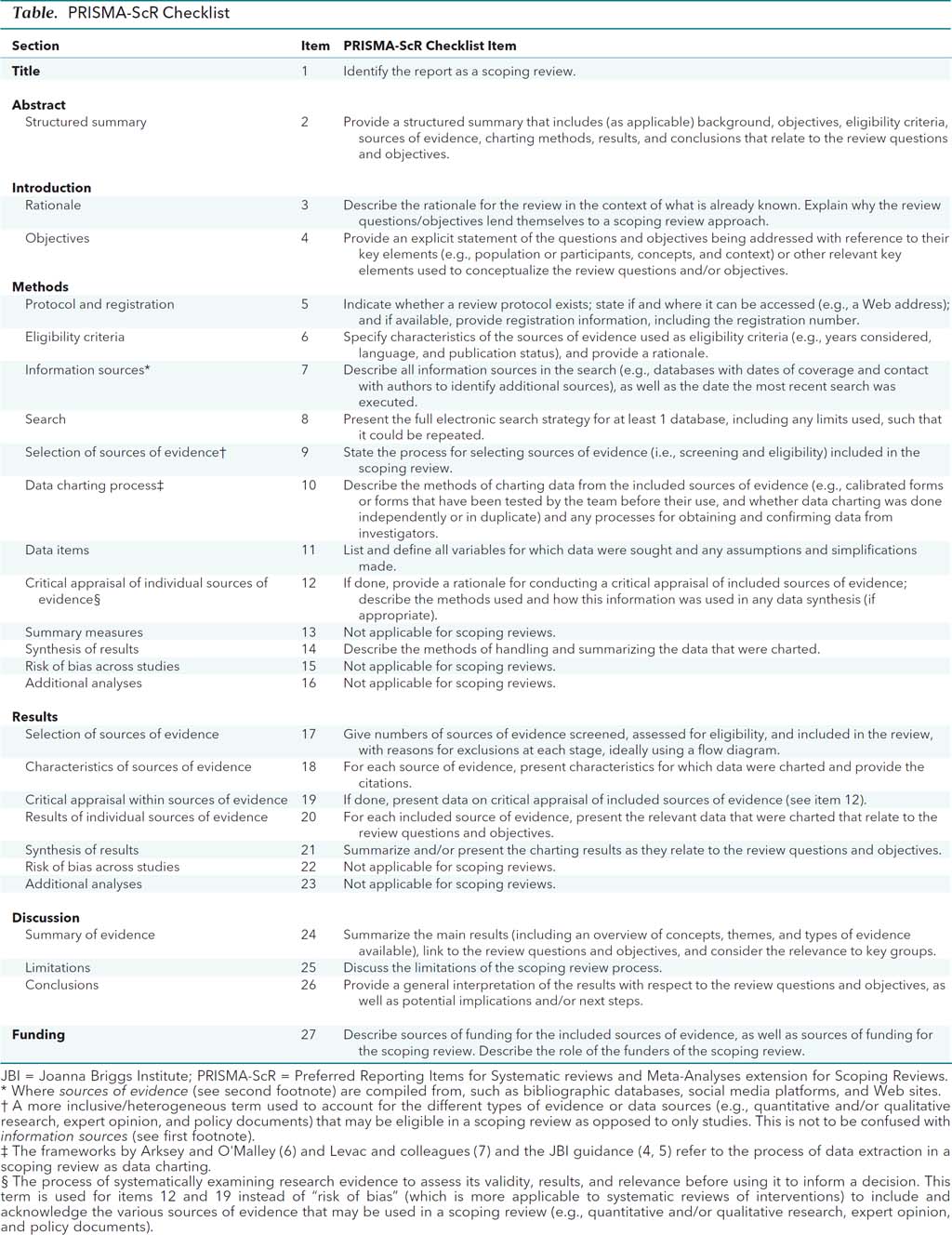


-From:Tricco AC, Lillie E, Zarin W, O'Brien KK, Colquhoun H, Levac D, Moher D, Peters MDJ, Horsley T, Weeks L, Hempel S, Akl EA, Chang C, McGowan J, Stewart L, Hartling L, Aldcroft A, Wilson MG, Garritty C, Lewin S, Godfrey CM, Macdonald MT, Langlois EV, Soares-Weiser K, Moriarty J, Clifford T, Tunçalp Ö, Straus SE. PRISMA Extension for Scoping Reviews (PRISMA-ScR): Checklist and Explanation. Ann Intern Med. 2018 Oct 2;169(7):467-473. doi: 10.7326/M18-0850
