# Supplementary material for: Educational strategies for enhancing medical students’ competency in laboratory medicine practice: a scoping review
Source: Front Med (Lausanne). 2026 May 11;13:1799809. doi: 10.3389/fmed.2026.1799809 (PMC13199127; doi:10.3389/fmed.2026.1799809)
Supplement: Supplementary file 2 [file Data_Sheet_2.pdf]

International Platform of Registered Systematic Review and Meta-analysis Protocols

INPLASY

INPLASY2025120034
doi: 10.37766/inplasy2025.12.0034
Received: 10 December 2025
Published: 10 December 2025

**Corresponding author:**
Yonggang Yang

43754962@qq.com

**Author Affiliation:**
Hangzhou Third People's Hospital.

# Educational Strategies for Enhancing Medical Students' Competency in Laboratory Medicine Practice: A Scoping Review

Yang, YG ; Chen, BB ; Tian, JY ; Chen, S ; Xu, W ; Lu, LJ ; Zhu, XR ; Liao, HQ ; Xu, SH.

**ADMINISTRATIVE INFORMATION**

**Support -** None.

**Review Stage at time of this submission -** Piloting of the study selection process.

**Conflicts of interest -** None declared.

**INPLASY registration number:** INPLASY20251200

**Amendments -** This protocol was registered with the International Platform of Registered Systematic Review and Meta-Analysis Protocols (INPLASY) on 10 December 2025 and was last updated on 10 December 2025.

## INTRODUCTION

**Review question / Objective** Review question：What educational intervention strategies, centered on clinical practice, have been reported in the literature to enhance the practical competency in laboratory medicine among clinical medical students? What is the landscape of these strategies in terms of their implementation contexts, methodological features, targeted competencies, and evidence of effectiveness?

Objective：

1. To identify and map the range of educational intervention strategies reported in the literature that aim to enhance clinical medical students' competency in laboratory medicine practice.
2. To analyze and describe the key characteristics of these strategies, with a focus on how they are anchored in "clinical practice," detailing their pedagogical approaches, implementation settings, and instructional activities.
3. To clarify the specific dimensions of laboratory medicine practice competency that these educational strategies target, and to synthesize a competency framework from the literature.
4. To synthesize the metho.

**Background** With the rapid advancement of medical technology, the role of laboratory medicine in clinical diagnosis, treatment, and prevention has become increasingly critical, with approximately 70% of medical decisions relying on test results. However, in medical education, the teaching of laboratory medicine receives limited attention. Current training in laboratory knowledge often suffers from a disconnect between theory and practice, fragmented curriculum content, and insufficient development of students' clinical



Yang et al. INPLASY protocol 2025120034. doi:10.37766/inplasy2025.12.0034 Downloaded from https://inplasy.com/inplasy-2025-12-0034/

reasoning skills. In the context of evolving precision medicine and integrated medicine, developing educational intervention strategies centered on clinical practice has become a sustained priority in medical education reform worldwide, aimed at enhancing the application of laboratory knowledge among clinical medical students.

**Rationale** Despite the critical role of laboratory medicine in clinical decision-making, it remains inadequately emphasized in medical education. This has resulted in deficiencies in both theoretical knowledge and practical application among medical students, ultimately impairing their ability to utilize laboratory data effectively in patient diagnosis and management. Consequently, there is a growing consensus on the need to enhance educational strategies centered on laboratory medicine practice. However, the existing literature on such strategies appears fragmented. Studies vary considerably in their methodologies, targeted competencies, instructional designs, and evaluation approaches, making it difficult to synthesize what has been done, what is effective, and where gaps persist. Therefore, a systematic scoping of these educational interventions is urgently required to map the current landscape. This scoping review aims to address this gap by systematically identifying, describing, and synthesizing the relevant evidence.

## METHODS

**Strategy of data synthesis** This scoping review will employ a structured, multi-stage process for data synthesis, aligned with established methodological frameworks. The primary aim is to conduct a descriptive and narrative synthesis to map the scope, nature, and key characteristics of the evidence, directly addressing the defined review objectives. The synthesis will not involve a meta-analysis or formal assessment of intervention effectiveness. The strategy consists of the following phases:
Phase 1: Data Charting and Management
A standardized data charting form will be developed in Microsoft Excel to systematically extract information from each included source. The form's variables are designed to capture data pertinent to the review objectives and will be piloted on a sample of studies. Key charting categories include:
Study Identification & Context: Author, year, country, study design/type, stated aims.
Population & Setting: Target learner group (e.g., undergraduate year, interns), educational and clinical context.
Intervention Characteristics: Description and classification of the educational strategy; pedagogical approach; mode of delivery (e.g., workshop, e-learning, bedside teaching); duration; integration with clinical practice.
Target Competencies: Explicitly stated or implied skills and knowledge domains in laboratory medicine (e.g., test selection, interpretation, understanding of pre-analytical factors).
Evaluation Methods: Study design for evaluation (e.g., pre-post test, survey); tools/instruments used; types of outcomes reported (e.g., knowledge, skills, attitudes, behaviors).
Reported Findings & Authors' Conclusions: Key results, identified strengths, limitations, and recommendations.
Phase 2: Descriptive Quantitative Analysis
Numerical and categorical data from the charting form will be analyzed descriptively to map the evidence landscape. This will involve calculating frequencies and generating visual summaries (tables, charts) to illustrate:
Temporal and geographical distribution of publications.
The frequency of different educational strategy types.
The distribution of study designs and outcome measures used for evaluation.
The prevalence of reported target competency domains.
Phase 3: Qualitative Thematic Synthesis and Narrative Summary
Textual and descriptive data will be analyzed thematically to identify patterns, relationships, and conceptual boundaries.
Intervention Categorization: Strategies will be inductively grouped into coherent categories based on shared primary characteristics (e.g., simulation-based, curriculum-integrated, digital tool-supported).
Analysis of Clinical Integration: Narrative data will be examined to synthesize common themes describing how interventions are anchored in or linked to clinical practice.
Competency Framework Development: Descriptions of target competencies will be coded and analyzed to identify, define, and relate core dimensions, informing a preliminary conceptual framework.
Synthesis of Contextual Factors: Reported facilitators, barriers, and implementation challenges will be extracted and categorized to identify common themes influencing the adoption and effectiveness of educational strategies.
Phase 4: Visual Mapping and Presentation of Results

Yang et al. INPLASY protocol 2025120034. doi:10.37766/inplasy2025.12.0034 Downloaded from https://inplasy.com/inplasy-2025-12-0034/

Findings from the quantitative and qualitative synthesis will be integrated to create structured overviews:
Evidence Matrices: Tabular formats will be used to juxtapose key characteristics (e.g., intervention categories vs. target competencies vs. evaluation methods), providing a clear map of connections and evidence gaps.
Conceptual Diagrams: A schematic figure will be developed to visually represent the main relationships between identified intervention types, their design features, associated competency targets, and evaluation approaches as derived from the synthesized literature.
Phase 5: Iterative Refinement and Team Validation
The synthesis is an iterative process. Initial analytic frameworks and theme development will be reviewed and refined by the research team. Regular consensus discussions will ensure consistent interpretation of data and that the synthesis accurately reflects the full scope of the included evidence. This collaborative approach enhances the trustworthiness and confirmability of the findings.
In summary, this synthesis strategy provides a rigorous, transparent, and reproducible plan for analyzing the extracted data. It is designed to produce a comprehensive descriptive account and visual map of the evidence, fulfilling the review's objectives to inform future educational practice, policy, and research in this domain.

**Eligibility criteria** This scoping review will use the PCC (Participants, Concept, Context) framework to define the inclusion criteria.
Participants: The review will include studies focusing on clinical medical students, defined as individuals enrolled in an undergraduate (pre-doctoral) medical degree program leading to a physician qualification. This may include students in classroom, clinical skills, and clerkship/rotational phases of their training. Studies focusing exclusively on postgraduate residents, fellows, practicing physicians, nursing students, or students of allied health professions (e.g., medical laboratory science) will be excluded.
Concept: The core concept is educational strategies aimed at developing competency in laboratory medicine practice. This includes:
Educational Strategies: Any described pedagogical intervention, approach, curriculum component, teaching method, or tool (e.g., specific courses, modules, workshops, e-learning, simulation, bedside teaching, clinical reasoning exercises).
Competency Focus: The strategy must explicitly aim to enhance knowledge, skills, or attitudes directly related to the clinical application of laboratory medicine. This includes, but is not limited to, test selection/appropriateness, result interpretation, diagnostic reasoning, understanding of pre-/post-analytical factors, and effective utilization of laboratory data in clinical decision-making. Studies focusing solely on theoretical knowledge of lab tests or basic pathophysiology without a clear link to clinical practice application will be excluded.
Context: The context is formal medical education settings. This encompasses educational activities delivered within medical schools, teaching hospitals, or affiliated clinical training sites as part of the core medical degree curriculum. Studies conducted in continuing professional development (CPD) or pure workplace training contexts for qualified staff will be excluded.

## Source of evidence screening and selection

This scoping review will use the PCC (Participants, Concept, Context) framework to define the inclusion criteria.
Participants: The review will include studies focusing on clinical medical students, defined as individuals enrolled in an undergraduate (pre-doctoral) medical degree program leading to a physician qualification. This may include students in classroom, clinical skills, and clerkship/rotational phases of their training. Studies focusing exclusively on postgraduate residents, fellows, practicing physicians, nursing students, or students of allied health professions (e.g., medical laboratory science) will be excluded.
Concept: The core concept is educational strategies aimed at developing competency in laboratory medicine practice. This includes:
Educational Strategies: Any described pedagogical intervention, approach, curriculum component, teaching method, or tool (e.g., specific courses, modules, workshops, e-learning, simulation, bedside teaching, clinical reasoning exercises).
Competency Focus: The strategy must explicitly aim to enhance knowledge, skills, or attitudes directly related to the clinical application of laboratory medicine. This includes, but is not limited to, test selection/appropriateness, result interpretation, diagnostic reasoning, understanding of pre-/post-analytical factors, and effective utilization of laboratory data in clinical decision-making. Studies focusing solely on theoretical knowledge of lab tests or basic pathophysiology without a clear link to clinical practice application will be excluded.
Context: The context is formal medical education settings. This encompasses educational activities delivered within medical schools, teaching hospitals, or affiliated clinical training sites as part of the core medical degree curriculum. Studies conducted in continuing professional development

Yang et al. INPLASY protocol 2025120034. doi:10.37766/inplasy2025.12.0034 Downloaded from https://inplasy.com/inplasy-2025-12-0034/

(CPD) or pure workplace training contexts for qualified staff will be excluded.
Source of Evidence Screening and Selection
The selection process will be conducted in three consecutive stages, managed using the systematic review software Covidence, and performed independently by two reviewers to ensure reliability.
Stage 1: Identification & Deduplication
All records retrieved from the database searches will be imported into Covidence. The software's automated function will identify and remove duplicate records.
Stage 2: Title and Abstract Screening
The two reviewers will independently screen the titles and abstracts of all unique records against the pre-defined PCC eligibility criteria. Records will be categorized as "include," "exclude," or "maybe." Any record marked for inclusion by either reviewer will proceed to the next stage.
Stage 3: Full-Text Review
The full texts of all records advanced from Stage 2 will be retrieved. The two reviewers will then independently assess each full-text article for final inclusion, applying the same eligibility criteria. At both screening stages, the reasons for exclusion will be documented.
Resolution of Disagreements
At the end of both Stage 2 and Stage 3, the results of the independent screenings will be compared. Any discrepancies between the two reviewers regarding the inclusion or exclusion of a source will be resolved through discussion. If a consensus cannot be reached, a third senior reviewer will be consulted to make the final decision. This process will be documented.

**Language restriction** Literature restriction will be applied, with searches limited to publications in English and Chinese.

**Country(ies) involved** China.

**Keywords** Laboratory medicine education; Medical students; Clinical competency; Educational strategies; Scoping review.

**Contributions of each author**
Author 1 - Yonggang Yang.
Email: 43754962@qq.com
Author 2 - Baobing Chen.
Author 3 - Jiyun Tian.
Author 4 - Song Chen.
Author 5 - Wei Xu.
Author 6 - Lijia Lu.
Author 7 - Xiurong Zhu.
Author 8 - Hongqiang Liao.
Author 9 - Shuihua Xu.

Yang et al. INPLASY protocol 2025120034. doi:10.37766/inplasy2025.12.0034 Downloaded from https://inplasy.com/inplasy-2025-12-0034/
