# Supplementary material for: Educational strategies for enhancing medical students’ competency in laboratory medicine practice: a scoping review
Source: Front Med (Lausanne). 2026 May 11;13:1799809. doi: 10.3389/fmed.2026.1799809 (PMC13199127; doi:10.3389/fmed.2026.1799809)
Supplement: Supplementary file 3 [file Data_Sheet_3.docx]

Supplementary material 3: Search strings for each data base

**Search string for CNKI:**

主题=(临床医学 + 医学教育 + 医学生 + 临床教学) AND 主题=(检验医学 + 医学检验 + 实验室诊断 + 临床检验) AND 主题=(教育 + 教学 + 课程 + 培训)

Translate to English：

subject=("clinical medicine" + "medical education" + "medical student" + "clinical teaching") AND subject=("laboratory medicine" + "medical laboratory science" + "laboratory diagnosis" + "clinical laboratory testing") AND subject=("education" + "teaching" + "curriculum" + "training")

**Search string for PubMed:**

("Clinical Medicine"[Mesh] OR "clinical medicine" OR "Medical Education"[Mesh] OR "medical education" OR "Students, Medical"[Mesh] OR "medical student*") AND

("Clinical Laboratory Techniques"[Mesh:exp] OR "Pathology, Clinical"[Mesh] OR "laboratory medicine" OR "clinical pathology" OR "clinical laboratory") AND

("Education, Medical"[Mesh] OR "educat*" OR "teach*" OR "train*" OR "curriculum" OR "competenc*")

**Search string for Chinese Medical Database (CMD):**

主题=(临床医学 OR 医学教育 OR 医学生) AND 主题=(检验医学 OR 医学检验 OR 临床检验) AND 主题=(教育 OR 教学 OR 培训)

Translate to English：

subject=("clinical medicine" OR "medical education" OR "medical student") AND subject=("laboratory medicine" OR "medical laboratory science" OR "clinical laboratory testing") AND subject=("education" OR "instruction" OR "training")

**Search string for Springer LINK:**

( ti:("clinical medicine" OR "medical education" OR "medical student") OR abs:("clinical medicine" OR "medical education" OR "medical student") ) AND

( ti:("laboratory medicine" OR "clinical pathology" OR "clinical laboratory") OR abs:("laboratory medicine" OR "clinical pathology" OR "clinical laboratory") ) AND

abs:(educat* OR teach* OR train* OR curriculum OR competenc*)

**Search string for Web of Science:**

TS=( ("clinical medicine" OR "medical education" OR "medical student*" OR "clinical clerkship" OR "clinical training") AND

("laboratory medicine" OR "clinical pathology" OR "Clinical Laboratory Techniques" OR "clinical laboratory science") AND

(educat* OR teach* OR train* OR curriculum OR "competency-based education"))
