# Supplementary material for: Educational strategies for enhancing medical students’ competency in laboratory medicine practice: a scoping review
Source: Front Med (Lausanne). 2026 May 11;13:1799809. doi: 10.3389/fmed.2026.1799809 (PMC13199127; doi:10.3389/fmed.2026.1799809)
Supplement: Supplementary file 4 [file Data_Sheet_4.docx]

Supplementary material 4

*Figure 1. PRISMA-ScR Flowchart of Literature Screening*

Identification

Records identified from other sources

(n = 3)

Including: Citation searching

Records identified through database searching (n =9,943)
Databases: PubMed, CNKI, CMA, Springer LINK, WOS

Duplicates removed

(n = 641)

Records identified

(n = 9,946)

Screening

Records excluded (n =9,067)
*Non-research records (n=3,352)
*Ineligible population (n=2,107)
*Non-practical educational intervention (n=2,207)
*Irrelevant to the topic (n=1401)

Records screened (title and abstract)

(n = 9,305)

Eligibility

Full-text articles excluded (n = 227)
*Ineligible population (n=101)
*Non-practical educational intervention (n=78)
*Irrelevant to the topic (n=43)
*Full text unavailable (n=3)
*Not in English or Chinese (n=2)

Full-text articles assessed for eligibility

(n = 238)

Included

Studies included in the review

(n = 11)

*Including: Quantitative studies (n = 11), Qualitative studies (n = 0)

*Notes:*

1. *This flowchart adheres to the PRISMA-ScR (Preferred Reporting Items for Systematic Reviews and Meta-Analyses extension for Scoping Reviews) statement.*
2. *The screening process was conducted independently by at least two reviewers. Discrepancies were resolved through discussion or consultation with a third reviewer.*
3. *Specific numbers (n = ) should be filled in according to the actual screening results.*

**Adapted from**：*Andrea C. Tricco, Erin Lillie, Wasifa Zarin, et al. PRISMA Extension for Scoping Reviews (PRISMA-ScR): Checklist and Explanation. Ann Intern Med.2018;169:467-473*
