# Supplementary material for: Educational strategies for enhancing medical students’ competency in laboratory medicine practice: a scoping review
Source: Front Med (Lausanne). 2026 May 11;13:1799809. doi: 10.3389/fmed.2026.1799809 (PMC13199127; doi:10.3389/fmed.2026.1799809)
Supplement: Supplementary file 5 [file Data_Sheet_5.docx]

**Supplementary material 5**

***Data Extraction Guide***

*This guide aims to ensure consistency among all members in understanding and recording key concepts when extracting data from literature in different languages, thereby eliminating ambiguity.*

**Framework of the Data Extraction Table and General Instructions**

**Tool:** Use a single Microsoft Excel file, creating a Independent Column for each included study.

**Working Language:** The recording language in the table is unified as English to facilitate international publication. However, extraction must be done against the original text, and key descriptions may retain citations from the original language.

**Extraction Principle:** Extract "what it is" (descriptive facts), not "how well it worked" (effectiveness conclusions). Information on effects belongs in the "Key Findings" column.

| **Field** | **Definition & Extraction Instructions** | **Key Points & Examples** |
| --- | --- | --- |
| **1. Study Identification & Context** | **Extract basic identifying and contextual information about the study.** |  |
| Authors (Year) | List main muthor. Year refers to the publication year. | e.g., Li et al. (2023); Wang Xuejing et al. (2018) |
| Country | The country where the study was conducted. | e.g., China, USA |
| Source Type | Judge based on study design and publication format. | Primary Research (reporting specific intervention and evaluation), Methodological Paper (describing curriculum development), Review . |
| Stated Aim/Purpose | Directly quote or precisely summarize the explicitly stated research objective or question from the text. | Extract from the "Objective" or "Background" section. e.g., "To explore the application effect of...", "To evaluate the effectiveness of the... teaching model", "To develop and assess...". |
| **2. Participants** | **Describe the target learner population of the study.** |  |
| Target Learner Group | Precisely describe the learner identity and stage of training. | e.g., "Clinical medicine interns", "Third-year clinical medicine undergraduates", "First-year medical students". Distinguish between "interns" and "clerkship students". |
| **3. Concept (Educational Strategy/Intervention)** | **Describe in detail the educational strategy or intervention being studied.** |  |
| Strategy Description | Summarize the core learning activity in 1-2 sentences, answering "What did the learners DO?". | Focus on actions. e.g., "Used a ... form for case interpretation practice", "Participated in small group discussion and analysis of a ... case". |
| Teaching & Learning Method | Induct the pedagogical method or paradigm used. | e.g., Case-Based Learning (CBL), Problem-Based Learning (PBL), Simulation-based education, Gamified learning, Interprofessional Education (IPE). |
| Mode of Delivery | Describe the specific operational format of the strategy. | e.g., Face-to-face workshop, Interactive online module, Laboratory clerkship rotation, Classroom-integrated session. |
| Duration/Intensity | Extract the total time or number of instructional hours of the intervention. | e.g., "A 2-credit-hour course", "A 4-week module", "A one-off 3-hour workshop". If not explicitly stated, record as "Not reported". |
| **4. Context** | **Describe the specific setting in which the educational intervention took place.** |  |
| Educational Setting | Specify the physical or organizational venue where the teaching occurred. | e.g., University medical school laboratory, Laboratory department of an affiliated teaching hospital, Online learning platform, Clinical skills center. |
| **5. Evaluation Methods** | **Describe how the effectiveness of the educational strategy was evaluated.** |  |
| Study Design | The empirical research design used to evaluate effectiveness. | e.g., Experimental studies (Randomized Controlled Trial (RCT), Non-randomized Controlled Trial (Quasi-experimental Study)); Observational studies (Descriptive Study, Analytical Study (e.g., Cross-sectional Survey)). |
| Tools Used | The specific instruments or methods used for data collection. | e.g., Knowledge test, Skill performance checklist, Likert-scale questionnaire, Focus group interview guide, Platform log data. |
| Type of Outcomes Reported | Categorize the measured competency domain(s) based on the evaluation tools. | e.g., Knowledge (test scores), Skills (interpretation accuracy, speed), Attitudes (satisfaction, confidence), Behaviour (engagement). |
| **6. Key Findings & Conclusions** | **Synthesize the reported results and authors' perspectives.** |  |
| Reported Key Results | From the "Results" section, distill objective findings directly related to the intervention. May include statistical significance. | e.g., "The post-test score of the intervention group was significantly higher than that of the control group (p<0.05)", "85% of students reported 'high' or 'very high' satisfaction with...". |
| Advantages/Limitations Noted by Authors | From the "Discussion" section, extract the authors' appraisal of the strategy itself or the research process. | e.g., "The tool provided immediate feedback, which was beneficial for error correction", "The small sample size of this study limits the generalizability of the findings". |
| Authors' Recommendations | Extract recommendations made by the authors for future practice or research based on this study. | e.g., "It is recommended to integrate the ... module into the core curriculum", "Future research needs to evaluate its long-term effects". |
